# CK5/6 and GATA3 Defined Phenotypes of Muscle-Invasive Bladder Cancer: Impact in Adjuvant Chemotherapy and Molecular Subtyping of Negative Cases

**DOI:** 10.3389/fmed.2022.875142

**Published:** 2022-06-16

**Authors:** Florestan J. Koll, Alina Schwarz, Jens Köllermann, Severine Banek, Luis Kluth, Clarissa Wittler, Katrin Bankov, Claudia Döring, Nina Becker, Felix K.H. Chun, Peter J. Wild, Henning Reis

**Affiliations:** ^1^Department of Urology, University Hospital Frankfurt, Goethe University, Frankfurt, Germany; ^2^Frankfurt Cancer Institute (FCI), University Hospital, Goethe University, Frankfurt, Germany; ^3^University Cancer Center (UCT) Frankfurt, University Hospital, Goethe University, Frankfurt, Germany; ^4^Dr. Senckenberg Institute of Pathology, University Hospital Frankfurt, Frankfurt, Germany; ^5^Frankfurt Institute for Advanced Studies, Frankfurt, Germany

**Keywords:** bladder cancer, molecular subtyping, immunohistochemistry, adjuvant chemotherapy, double negative, consensus classification

## Abstract

**Introduction and Objective:**

Identifying patients that benefit from cisplatin-based adjuvant chemotherapy is a major issue in the management of muscle-invasive bladder cancer (MIBC). The purpose of this study is to correlate “luminal” and “basal” type protein expression with histological subtypes, to investigate the prognostic impact on survival after adjuvant chemotherapy and to define molecular consensus subtypes of “double negative” patients (i.e., without expression of CK5/6 or GATA3).

**Materials and Methods:**

We performed immunohistochemical (IHC) analysis of CK5/6 and GATA3 for surrogate molecular subtyping in 181 MIBC samples. The mRNA expression profiles for molecular consensus classification were determined in CK5/6 and GATA3 (double) negative cases using a transcriptome panel with 19.398 mRNA targets (HTG Molecular Diagnostics). Data of 110 patients undergoing radical cystectomy were available for survival analysis.

**Results:**

The expression of CK5/6 correlated with squamous histological subtype (96%) and expression of GATA3 was associated with micropapillary histology (100%). In the multivariate Cox-regression model, patients receiving adjuvant chemotherapy had a significant survival benefit (hazard ratio [*HR*]: 0.19 95% confidence interval [*CI*]: 0.1–0.4, *p* < 0.001) and double-negative cases had decreased OS (*HR*: 4.07; 95% *CI*: 1.5–10.9, *p* = 0.005). Double negative cases were classified as NE-like (30%), stroma-rich (30%), and Ba/Sq (40%) consensus molecular subtypes and displaying different histological subtypes.

**Conclusion:**

Immunohistochemical-based classification was associated with histological subtypes of urothelial MIBC. IHC markers like CK5/6 and GATA3 that are used in pathological routine could help to identify patients with basal and luminal tumor characteristics. However, a two-sided classification system might not sufficiently reflect the heterogeneity of bladder cancer to make treatment decisions. Especially the group of IHC-double negative cases, as further analyzed by mRNA expression profiling, are a heterogeneous group with different implications for therapy.

## Introduction

Bladder cancer (BCa) is the second most common genitourinary malignancy with about 570,000 new cases worldwide every year (1). About 25% of patients present with the muscle-invasive disease at the time of diagnosis and >90% of cases are urothelial carcinomas. The current standard of care for muscle invasive BCa (MIBC) is radical cystectomy with pelvic lymphadenectomy. However, relapse rates after surgery are high and 5-year overall survival (*OS*) rates are about 43% for pT3-tumors and 25% if the tumor has spread to local lymph nodes (2, 3). Cisplatin-based adjuvant chemotherapy may prolong survival rates and should be offered to patients with pT3/4 and/or pN + tumors (4). Patient selection for adjuvant chemotherapy is based on pathological tumor stage and chemo-eligibility, but no biomarker-based selection criteria for cisplatin-based chemotherapies exist or are included in current guidelines (4). The predictive biomarker-based decisions are needed to identify potential responders to chemotherapy and those that would be unnecessarily exposed to adjuvant therapy toxicities.

In recent years, genomic sequencing techniques have advanced, leading to comprehensive genomic characterization of BCa cohorts. This led to transcriptomic-based molecular subtyping of cancers and drove our understanding of BCa biology (5–9). It has been proposed that patients with basal tumors benefit most of neoadjuvant chemotherapy (NAC), whereas luminal tumors have a better prognosis regardless of the application of NAC (10, 11). However, contradictory results have been published and different nomenclatures, definitions, numbers of molecular subtypes, and inter-/intra-tumoral heterogeneity of BCa have hindered prospective validation and clinical translation.

To facilitate the clinical implementation of subtyping into clinical routine, immunohistochemical (IHC) markers that refer to “basal” and “luminal” molecular subtypes have been proposed (12–14). Guo et al. reported that IHC-staining with GATA3 and CK5/6 can classify the BCa correctly in over 80% of the cases into luminal and basal molecular subtypes. However, it remains unclear to which molecular subtype tumors without GATA3, nor CK5/6 expression (double negative) can be assigned.

In the present study, we explore IHC markers as surrogate markers for molecular subtyping, correlations with histologic subtypes, and impact on survival with adjuvant chemotherapy in a mono-institutional cystectomy cohort of urothelial MIBC. In addition, we performed RNA-sequencing of the group of “double negative” cases (CK5/6 and GATA3 negative), which are a heterogenous group on the molecular level.

## Materials and Methods

### Cohort

Tissue/tumor samples and patient data used in this study were provided by the University Cancer Center Frankfurt (UCT). The written informed consent was obtained from all patients and the study was approved by the institutional review boards of the UCT and the ethical committee at the University Hospital Frankfurt (project-number: SUG-6-2018 and UCT-53-2021) which was conducted according to local and national regulations and according to the Declaration of Helsinki.

A total of 186 FFPE tissue samples from 181 patients with MIBC treated at the Department of Urology, University Hospital Frankfurt from 2010 to 2020 were retrieved from the archive of the Senckenberg Biobank of the Senckenberg Institute of Pathology.

Clinico-pathological and follow-up data were gathered from medical charts and records of the University Cancer Center and independently reviewed by two authors.

Histopathology of all cases was systematically re-reviewed by two experienced genitourinary pathologists according to current WHO-criteria (15). Histological subtypes were reported if at least 10% of tumor showed subtype histology including pure and mixed tumors.

### Immunohistochemical Analysis

For construction of the tissue microarray (TMA), one tissue core (diameter 1 mm) of a representative tumor area was taken from a “donor” block and was arranged in a new “recipient” block using the TMA Grandmaster (3DHISTECH, Budapest, Hungary).

Hematoxylin and eosin stain slides were automatically developed on a Tissue-Tek Prisma Plus staining device (Sakura Finetek, Torrance, CA, United States). All IHC-analyses were conducted using the DAKO Omnis staining system (Agilent, Santa Clara, CA, United States) with the DAKO FLEX-Envision Kit (Agilent) according to manufacturer’s instruction. We performed staining of CK5/6 (Clone: D5/16 B4; ready-to-use kit; Dako/Agilent, Santa Clara, CA, United States) and GATA3 (Clone: L50-823; ready-to-use kit; Cell Marque, Rocklin, CA, United States). IHC “double negative” was defined as negative for expression of CK5/6 and GATA3 and “double positive” was defined as positive for expression of CK5/6 as well as GATA3.

Stained slides were scanned with the Pannoramic slide scanner (3DHISTECH, Budapest, Hungary). The quantitative analysis of IHC was annotated by two genitourinary pathologists. TMA cores with either absence of representative tumor tissue or presence of staining artifacts were excluded from the analysis.

### RNA Isolation and Molecular Subtype Calling

A 1 mm punch was taken from the FFPE blocks of a representative tumor area with at least 50% tumor content. RNA was isolated using the “truXTRAC FFPE total NA Kit–Column” (Covaris, Woburn, MA, United States) and RNA-concentration was measured by using the QuantiFluor RNA System (Promega, Madison, WI, United States) according to the manufacturer’s protocol. The mRNA expression of 19,398 mRNA targets was determined using the HTG Transcriptome Panel (HTG Molecular Diagnostics, Tucson, AZ, United States) on Illumina NextSeq 550 system (Illumina, San Diego, CA, United States). Gene counts were normalized using median normalization and log2-transformed for further analysis. Sequencing data have been uploaded to the Gene Expression Omnibus (GSE198607). IHC double negative samples were defined as negative for CK5/6 and GATA3 in IHC analyses and classified according to the six molecular consensus classes of MIBC using the R-based consensus MIBC classification tool and the Bioconductor-package for R (6).

### Statistical Analysis

We performed descriptive statistics of all data.

For the survival analysis, only patients with radical cystectomy in “curative intent” (*n* = 110) were included to create a homogenous cohort. In total, 71 patients that did not fulfill this requirement were excluded, i.e., no radical cystectomy, with primary metastatic disease, NAC or missing follow up data. We defined the *OS* as main endpoint of interest, which was defined as time interval between surgery and death. Secondary endpoint was disease-free survival (*DFS*), defined as time interval between surgery and death due to BCa or recurrence.

Kaplan–Meier method was used to estimate and illustrate survival probabilities. We used uni- and multivariable Cox’s proportional hazards models to estimate the hazard ratio (*HR*) and corresponding 95% confidence interval (*CI*) for covariates for OS and DFS. All tests were two-tailed, and a significance level of α = 5% was used. The statistical analyses were performed using the R Statistical Software (Version 4.1) and R Studio (Version 2021.09.1 + 372).

## Results

### Patient Characteristics

Overall, we included specimen of 181 patients with MIBC on the TMA. Samples were obtained from transurethral resection of the bladder (TURB) in 86 (47.5%) cases and from cystectomy in 95 (52.5%) cases. The median age was 71 years (*IQR*: 62–78). A total of 140 (77%) patients were male and 41 (23%) were female. The clinico-pathological details of the cohort are summarized in Table 1.

**TABLE 1:** Clinico-pathological details of 181 patients on the TMA analyzed for histological subtype of urothelial carcinoma and immunohistochemistry.

Median Age (IQR)		71 (62–78)
**Gender**	Male	140 (77%)
	Female	41 (23%)
**Max. tumor-Stage**	pT2	87 (48%)
	pT3	67 (37%)
	pT4	27 (15%)
**Histological subtype on TMA spot**	NOS	131 (72%)
	Squamous	25 (14%)
	Micropapillary	9 (5%)
	Neuroendocrine	4 (2%)
	Sarcomatoid	3 (2%)
	Plasmacytoid	3 (2%)
	Other (1 lymphoepithelial, 1 clear cell, 1 glandular, 3 giant cell)	6 (3%)

*IQR, interquartile range; NOS, not otherwise specified; TMA, tissue micro array.*

### Immunohistochemical-Classification

**FIGURE 1:**
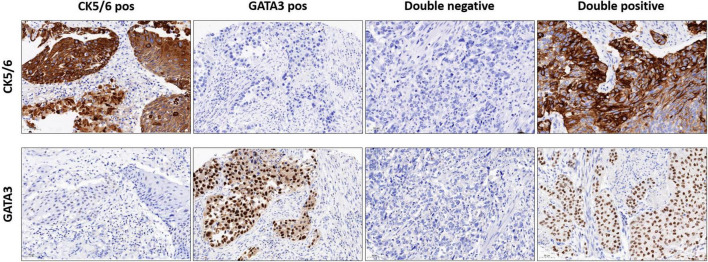
Representative images of IHC staining of CK5/6 positive, GATA 3 positive, double negative, and double positive cases (magnification 200 ×).

176 TMA-Spots were evaluable for the IHC-status of CK5/6 and GATA 3. Cases were classified into CK5/6 positive, GATA3 positive, “double negative” or “double positive” (representative images in Figure 1). CK5/6 positive cases were significantly associated with female gender (63% of tumors of female patients had CK5/6 expression vs. 37% tumors of male patients had CK5/6 expression, *p* = 0.004). CK5/6 positivity correlated with squamous histological subtype (24 of 25 cases [96%] had positive CK5/6 expression), whereas all 9 micropapillary cases were negative for the basal marker CK5/6 and positive for GATA 3 (*p* < 0.0001). All cases with neuroendocrine subtype were double negative. Twelve cases with other histological subtypes were negative for CK5/6, positive for GATA3 in 6 cases (one sarcomatoid, three plasmacytoid, one undifferentiated [giant cell], one glandular), double negative in 4 cases (one clear cell, one sarcomatoid, one undifferentiated [giant cell], one lymphoepithelial), and double positive in 2 cases (one sarcomatoid, one undifferentiated [giant cell]). Correlation of gender, tumor stage, age, and histology subtype with the two IHC markers are shown in Table 2.

**TABLE 2:** Association of clinic-pathological findings with the IHC-markers CK5/6 and GATA3.

		*n* (%)	CK5/6 positive (*n* = 32)	GATA3 positive (*n* = 81)	Double negative (*n* = 17)	Double positive (*n* = 46)	*p*
**Age**	**<70**	73 (41%)	15 (47%)	29 (36%)	9 (53%)	20 (44%)	*0.5*
	**≥70**	103 (59%)	17 (53%)	52 (64%)	8 (47%)	26 (15%)	
**Gender**	**Male**	135 (77%)	23 (72%)	67 (83%)	16 (94%)	29 (63%)	***0.021***
	**Female**	41 (23%)	9 (28%)	14 (17%)	1 (6%)	17 (37%)	
**Max. Tumor-Stage**	**pT2**	83 (47%)	11 (34%)	45 (56%)	8 (47%)	19 (41%)	*0.138*
	**pT3**	66 (38%)	18 (56%)	23 (28%)	5 (29%)	20 (44%)	
	**pT4**	27 (15%)	3 (10%)	13 (16%)	4 (24%)	7 (15%)	
**Histological subtype**	**NOS**	127 (72%)	18 (59%)	65 (80%)	10 (59%)	34 (74%)	**<*0.0001***
	**Squamous**	25 (14%)	14 (44%)	1 (1%)	0	10 (22%)	
	**Micropapillary**	9 (5%)	0	9 (11%)	0	0	
	**Neuroendocrine**	3 (2%)	0	0	3 (18%)	0	
	**Sarcomatoid**	3 (2%)	0	1 (1%)	1 (6%)	1 (2%)	
	**Plasmacytoid**	3 (2%)	0	3 (4%)	0	0	
	**Undifferentiated (giant cell)**	3 (2%)	0	1 (1%)	1 (6%)	1 (2%)	
	**Glandular**	1 (0.6%)	0	1 (1%)	0	0	
	**Clear cell**	1 (0.6%)	0	0	1 (6%)	0	
	**Lymphoepithelial**	1 (0.6%)	0	0	1 (6%)	0	

*A total of 176 spots were evaluated. The p-values were calculated using Pearson–Chi square test. NOS, not otherwise specified. Bold values indicate significant differences with p-values < 0.05.*

### Survival Analysis

We assessed survival rates of 110 patients with adequate follow up that received radical cystectomy in curative intend. Median follow-up was 66 months (*IQR*: 34–98 months). In this group, 35 patients received at least two cycles of adjuvant chemotherapy. Table 3 shows patients characteristics, tumor stage, and IHC markers for patients with and without adjuvant chemotherapy.

**TABLE 3:** Characteristics of patients with and without adjuvant chemotherapy.

		CE only *n* = 75	Adjuvant Chemotherapy *n* = 35	*p*
**Age (IQR)**		72 (64.25–76.75)	60 (52.75–71)	
**Age**	<71	32 (43%)	26 (74%)	***0.002***
	≥71	43 (57%)	9 (26%)	
**Gender**	Male	59 (79%)	28 (80%)	*1.0*
	Female	16 (21%)	7 (20%)	
**Stage Max.**	pT2	23 (31%)	5 (14%)	*0.12*
	pT3	37 (49%)	24 (69%)	
	pT4	15 (20%)	6 (17%)	
**Lymph node status**	pN0	42 (56%)	13 (37%)	*0.084*
	pN1 + pNx	33 (44%)	22 (63%)	
**R-Status**	R0	60 (80%)	28 (80%)	*1.0*
	R1/R2/Rx	15 (20%)	7 (20%)	
**Histological subtype**	NOS	57 (77%)	23 (66%)	*0.39*
	Squamous	8 (11%)	4 (11%)	
	Micropapillary	5 (7%)	2 (6%)	
	Neuroendocrine	1 (1%)	2 (6%)	
	Other	3 (4%)	4 (11%)	
**Type of chemotherapy**	Gem/Cis		28 (80%)	
	Gem/Carbo		4 (11%)	
	Platin/Etoposid		2 (6%)	
	other		1 (3%)	
**Recurrence**	No	54 (72%)	18 (51%)	*0.058*
	Yes	21 (28%)	17 (49%)	
**CK5/6 + GATA3**	CK5/6 pos	14 (19%)	5 (15%)	*0.45*
	GATA 3 pos	36 (48%)	13 (38%)	
	Double neg	7 (9%)	6 (18%)	
	Double pos	18 (24%)	10 (29%)	

*CE, cystectomy; NOS, not otherwise specified; Gem/Cis, gemcitabine/cisplatin; Gem/Carbo, gemcitabine/carboplatin; R-Status, resection status. Bold values indicate significant differences with p-values < 0.05.*

**FIGURE 2:**
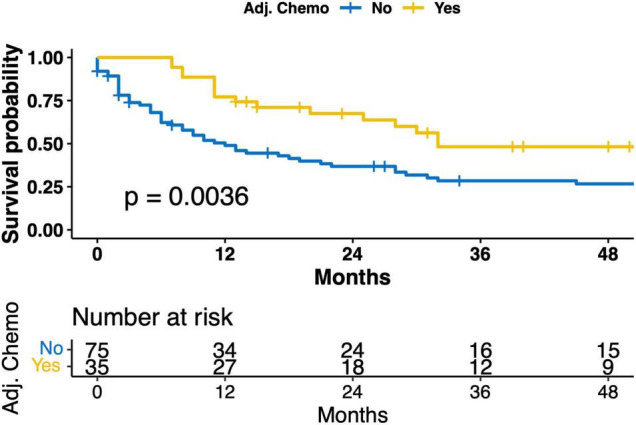
Kaplan–Meier curve for overall survival (OS) for patients with and without adjuvant chemotherapy.

Tumor and lymph node stage as well as the application of adjuvant chemotherapy were significantly associated with OS (Figure 2 and Supplementary Figures 1, 2). The 12-month OS and DFS rates were 49% and 41 without and 77 and 62% with adjuvant chemotherapy, respectively.

**FIGURE 3:**
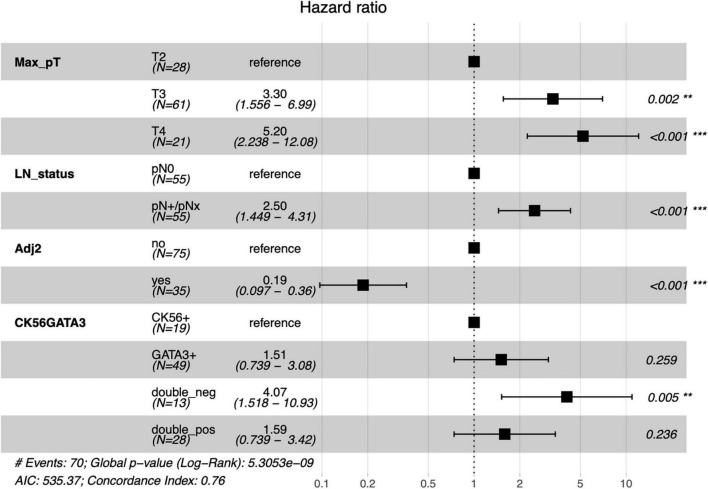
Multivariate cox-regression model for OS adjusting for tumor and LN stage, adjuvant chemotherapy, and the IHC-markers CK5/6 and GATA3. Number of events: 70; Global *p*-value (Log Rank): 5.3053e-09. LN, lymph node; Adj2, at least two cycles of adjuvant chemotherapy. ^**^p < 0.05; ^***^p < 0.001.

In the total cohort, neither IHC markers (CK5/6, GATA3) nor the histological subtype were significantly associated with OS or DFS (Table 4 and Supplementary Figure 3). After stratification for patients receiving only the cystectomy vs. patients receiving an adjuvant chemotherapy, expression of CK5/6 had a *HR* of 0.4 (95% *CI*: 0.2–1.2, *p* = 0.09) in the adjuvant chemotherapy group. A multivariate cox-regression model adjusting for tumor and lymph node (LN) stage, adjuvant chemotherapy, and IHC-staining is shown in Figure 3. In addition to tumor- and LN stage, the double negative cases were associated with an increased risk of death (*HR*: 4.07; 95% *CI*: 1.5–10.9, *p* = 0.005). Adjuvant chemotherapy was associated with the survival benefit (*HR*: 0.19; 95% *CI*: 0.10–0.36, *p* < 0.001). The multivariate cox-regression model for DFS is shown in the Supplementary Material.

**TABLE 4:** Univariate cox-regression model for histological subtype and IHC-markers CK5/6 and GATA3 stratified for patients with and without adjuvant chemotherapy.

		Total cohort (*n* = 110)	*p*	Adjuvant chemotherapy (*n* = 35)	*p*	CE only (*n* = 75)	*p*
**Histological subtype on TMA**	NOS	Reference		Reference		Reference	
	Squamous	0.8 (0.3–1.7)	*0.5*	0.4 (0.06–3.4)	*0.6*	0.9 (0.4–2.2)	*0.9*
	Micropapillary	1.2 (0.5–3.1)	*0.7*	2.9 (0.6–13.1)	*0.2*	0.89 (0.3–2.9)	*0.9*
	Neuroendocine	0.9 (0.2–4.1)	*0.9*	1.8 (0.2–14.1)	*0.6*	NA	
	Other	0.7 (0.3–1.9)	*0.5*	0.9 (0.2–4.2)	*0.9*	0.8 (0.2–3.5)	*0.8*
**CK56**	Pos	0.9 (0.5–1.4)	*0.5*	0.4 (0.2–1.2)	*0.09*	1.1 (0.7–1.9)	*0.7*
**GATA3**	Pos	0.8 (0.5–1.3)	*0.3*	0.7 (0.3–1.8)	*0.4*	0.8 (0.4–1.4)	*0.4*
**CK56 + GATA3**	CK56 pos	Reference		Reference			
	GATA3 pos	1.0 (0.5–2.0)	*0.9*	1.9 (0.4–8.8)	*0.4*	0.9 (0.4–1.8)	*0.71*
	Double neg	1.6 (0.7–3.7)	*0.3*	0.3 (0.5–16.7)	*0.2*	1.7 (0.6–4.7)	*0.31*
	Double pos	1.0 (0.5–2.1)	*1.0*	1.1 (0.1–5.0)	*0.8*	1.3 (0.6–2.8)	*0.59*

*CE, cystectomy; NOS, not otherwise specified.*

### Molecular Analysis of CK5/6 and GATA3 Negative Cases

**FIGURE 4:**
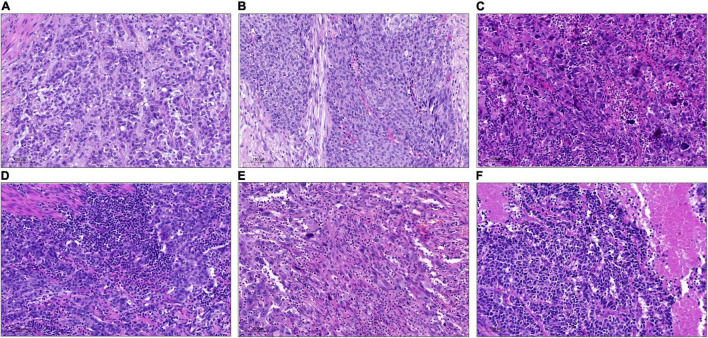
Representative pictures of different histological subtypes on the whole slides classified as double negative in tissue micro array (TMA)-analysis and their molecular consensus subtypes. **(A)** Histological subtype: NOS; molecular Subtype: Stroma-rich. **(B)** Histological subtype: NOS (+ squamous on whole slide); molecular subtype: Ba/Sq. **(C)** Histological subtype: poorly differentiated/giant cell; molecular subtype: Ba/Sq. **(D)** Histological subtype: lymphoepithelial; molecular subtype: Ba/Sq. **(E)** Histological subtype: sarcomatoid; molecular subtype: Ba/Sq. **(F)** Histological subtype: neuroendocrine; molecular subtype: NE-like. Ba/Sq, basal/squamous; NE, neuroendocrine; NOS, not otherwise specified.

Ten double negative cases were analyzed using the HTG Transcriptome Panel and molecular subtypes were determined according to the consensus classification of Kamoun et al. (6). Three cases were classified as stroma-rich, three as NE-like and four as Ba/Sq. Table 5 shows molecular subtypes with histological subtype and pathological stage. The Ba/Sq cases showed heterogeneous histology with lymphoepithelial, undifferentiated (giant cell) and sarcomatoid subtypes. According to the UNC classification system, all cases were classified as basal (8). And according to the TCGA classification system, cases were classified as basal-squamous and neuronal (5). Representative HE-stained pictures of histological subtype and their molecular subtype are shown in Figure 4.

**TABLE 5:** Description of double negative cases analyzed for mRNA expression profiles, including histological subtypes on TMA and whole slide, and molecular subtypes according to the consensus, UNC, and TCGA (5) classifier.

Patient	Age at surgery	Gender	Max. pT stage	LN metastases	Predominant histological subtype on TMA	Histological subtype on whole slide	Adjuvant chemotherapy	Consensus class	UNC subtype	TCGA subtype
001_069	68	m	3b	Yes	Lymphoepithelial	Lymphoepithelial	Yes	Ba/Sq	Basal	Basal_squamous
001_004	71	m	4a	No	Undifferentiated/Giant cell	Undifferentiated/Giant cell	No	Ba/Sq	Basal	Basal_squamous
001_033	71	m	3b	Yes	NOS	NOS + Squamous	No	Ba/Sq	Basal	Basal_squamous
001_039	60	m	2b	Yes	Sarcomatoid	Sarcomatoid	Yes	Ba/Sq	Basal	Basal_squamous
001_012	57	m	4b	Yes	NOS	NOS	Yes	Stroma-rich	Basal	Basal_squamous
001_040	78	m	4a	Yes	NOS	NOS	Yes	Stroma-rich	Basal	Basal_squamous
001_065	70	m	4a	No	NOS	NOS + Pseudoglandular	No	Stroma-rich	Basal	Neuronal
001_021	62	m	3b	No	NOS	Neuroendocrine	Yes	NE-like	Basal	Neuronal
001_050	62	m	3b	Yes	Neuroendocrine	Neuroendocrine	Yes	NE-like	Basal	Neuronal
001_071	67	m	2b	No	Neuroendocrine	Neuroendocrine	No	NE-like	Basal	Neuronal

*Ba/Sq, basal/squamous; m, male; NE, neuroendocrine; NOS, not otherwise specified; UNC, University of North Carolina; TCGA, the cancer genome atlas.*

## Discussion

This study shows a high concordance of an IHC-subclassification based on the two markers CK5/6 and GATA3 with histological subtypes in MIBC. Double negative patients without expression of CK5/6 nor GATA3 had decreased *OS* rates. Subtyping of double negative cases revealed a histological and molecular heterogeneous subgroup. Strengths of our study are the pathological re-review of all cases, evaluation of standardized IHC-staining, the use of a broad transcriptome panel for molecular phenotyping, and the analysis of adjuvant chemotherapy in a clinically well-annotated cohort of patients with MIBC.

The use of molecular subtyping to guide the selection of systemic therapies has been proposed for NAC and immune checkpoint inhibitors (7, 10, 16). However, contradictory results published in the past years and the diversity of molecular subtype taxonomy until the publication of the consensus classification have hindered the use and clinical translation (6, 17–19). Data to provide guidance to select patients for adjuvant chemotherapy based on molecular subtypes are sparse.

It has been suggested that CK5/6 and GATA3 expression can identify basal and luminal molecular subtypes in 80–90% of cases which could be a useful tool in routine pathological assessments to identify the basic molecular subtypes of BCa (12). Other studies using more markers confirmed a good correlation of IHC-based subtyping with gene expression-based subtypes (determined by targeted NanoString panels), but still with a risk of differing classification in about 15–20% of cases (13, 20).

Our results show that CK5/6 expression was associated with squamous histological subtype and female gender, which is in concordance with published data (6, 13). The same applies to the positive correlation between GATA3-expression and micropapillary histological subtype. Four cases with neuroendocrine histological subtype were double negative in IHC-analyses.

To test the two-marker-based IHC-classification for its predictive value of chemotherapy efficacy, we performed analyses in the cohort of adjuvant-treated patients. We are aware, that our survival analyses, which demonstrated a significant benefit for patients receiving adjuvant chemotherapy, hold the risk of selection bias since patients receiving chemotherapy were younger and kidney function or comorbidities were not considered. However, when analyzing only patients receiving chemotherapy, none of the markers predicted survival, but cases expressing the basal marker CK5/6 tend to have improved survival with adjuvant chemotherapy, with the level of significance missed (*HR* 0.42 95% *KI* 0.15–1.2, *p* = 0.09). These results are in line with the available data for NAC proposing that patients with basal-like tumors respond better to chemotherapy, whereas luminal-like tumors have an inferior response to chemotherapy.

For the neoadjuvant setting, Seiler and colleagues developed a single-sample genomic subtyping classifier to subdivide patients into four groups revealing that patients with basal tumors had the most improvement in OS with NAC compared with surgery alone (10). More recent studied showed similar results for non-luminal tumors (11, 20). Contrary results from Sjödahl and colleagues as well as Taber and colleagues, however, indicated that basal tumors less frequently respond to NAC and according to Kamoun and colleagues, the consensus molecular subtypes do not correlate with response to NAC (6, 17, 18). Therefore, further research will have to clarify the role of molecular subtypes as predictive markers of chemotherapy efficacy.

In addition to the findings in cases with IHC expression of at least one marker, we detected a subgroup of double negative cases without expression of CK5/6 or GATA3. The cohort seems to be of biological significance, as patients in this group had an increased risk of death in multivariate analysis. This finding has recently been described also by other groups (13, 14). However, no detailed molecular analyses were reported, as no full transcriptomic analyses were performed (12, 13). We, therefore, conducted further molecular analyses of the double negative group using an mRNA transcriptome panel covering approximately 19,300 targets, thus enabling us to call consensus class molecular subtypes.

Double negative cases tended to be molecularly basal-like according to the UNC classifier but are more heterogenous than a two tailed-classification can represent. Three molecular subtypes (Ba/Sq, stroma-rich, neuronal) and five histological subtypes (NOS, neuroendocrine, sarcomatoid, undifferentiated/giant cell, lymphoepithelioma-like) were present in this group. This highlights the heterogeneity of double negative cases and implicates that a more complex system than a two-tailed classification might be needed for more individualized treatment decisions. For example, the (molecular and histological) neuroendocrine subtype is known to have a poor prognosis and thus should be treated aggressively with upfront chemotherapy and might be responsive to immune checkpoint inhibition (4, 16). On the other hand, the lymphoepithelioma-like subtype of urothelial carcinoma is associated with a more favorable prognosis and might be more responsive to immune checkpoint inhibition as proposed by the results of the PURE-1 trial (21, 22). The well-known fact of intra-tumoral heterogeneity in urothelial BCa adds to these facts.

The limitations of our study are the retrospective design and the low number of molecular analyses. Due to low numbers, we did not include patients receiving NAC in our survival analysis. Selecting patients for NAC, which is the recommended treatment for MIBC, might be of higher clinical relevance than for adjuvant chemotherapy. In addition, we performed two IHC-marker analyses only to surrogate basal and luminal molecular subtypes. However, this was intentionally done to create comparability with the literature. Other studies using more markers for protein-based subtyping reported similar results with high concordance of histological subtypes and IHC, but also demonstrated that protein-based subtyping did not predict survival in population-based cystectomy cohorts (13, 19). Thus, the question remains if a limited protein-based assessment of surrogate markers for molecular subtypes can serve as a predictor for chemotherapy response or if mRNA-expression profiles are necessary for adequate determination of molecular subtypes or other markers like immune cell infiltration are necessary for patient selection (18). So far, the histopathological staging remains the most important prognostic factor (23). However, efforts to bring the results of the large transcriptomic studies into a clinical routine are underway (24).

In conclusion, we demonstrated that an IHC-based classification was associated with histological subtypes of urothelial MIBC. Although IHC markers used in pathological routine might help to identify patients with basal and luminal tumor characteristics, a two-sided classification system might not sufficiently reflect the heterogeneity of BCa to guide treatment decisions. Especially the group of IHC-double negative cases is a heterogeneous group with different implications for therapy.

## Data Availability Statement

The datasets presented in this study can be found in online repositories. The names of the repository/repositories and accession number(s) can be found below: The gene expression omnibus (GEO) with accession GSE198607 (https://www.ncbi.nlm.nih.gov/geo/query/acc.cgi?acc=GSE198607).

## Ethics Statement

Written informed consent was obtained from all patients, and the study was approved by the Institutional Review Boards of the UCT and the Ethical Committee at the University Hospital Frankfurt (project numbers: SUG-6-2018 and UCT-53-2021).

## Author Contributions

FK, HR, and PW contributed to conception and design of the study. FK, LK, SB, CW, AS, and KB organized the database and samples. AS, NB, KB, JK, HR, and PW performed data curation and formal analysis. FK and CD performed the statistical analysis and analysis of sequencing data. AS, NB, and KB performed IHC. FK wrote the first draft of the manuscript. HR, PW, and FC performed review and editing of the manuscript and supervision. All authors contributed to manuscript revision, read, and approved the submitted version.

## Conflict of Interest

Sequencing was performed as part of a collaboration agreement with HTG Molecular Diagnostics. The authors declare that the research was conducted in the absence of any commercial or financial relationships that could be construed as a potential conflict of interest.

## Publisher’s Note

All claims expressed in this article are solely those of the authors and do not necessarily represent those of their affiliated organizations, or those of the publisher, the editors and the reviewers. Any product that may be evaluated in this article, or claim that may be made by its manufacturer, is not guaranteed or endorsed by the publisher.
