# Supplementary material for: CK5/6 and GATA3 Defined Phenotypes of Muscle-Invasive Bladder Cancer: Impact in Adjuvant Chemotherapy and Molecular Subtyping of Negative Cases
Source: Front Med (Lausanne). 2022 Jun 16;9:875142. doi: 10.3389/fmed.2022.875142 (PMC9243590; doi:10.3389/fmed.2022.875142)
Supplement: Supplementary file 1 [file Data_Sheet_1.pdf]

Supplementary Material

CK5/6 and GATA3 defined phenotypes of muscle-invasive bladder cancer: impact in adjuvant chemotherapy and molecular subtyping of negative cases

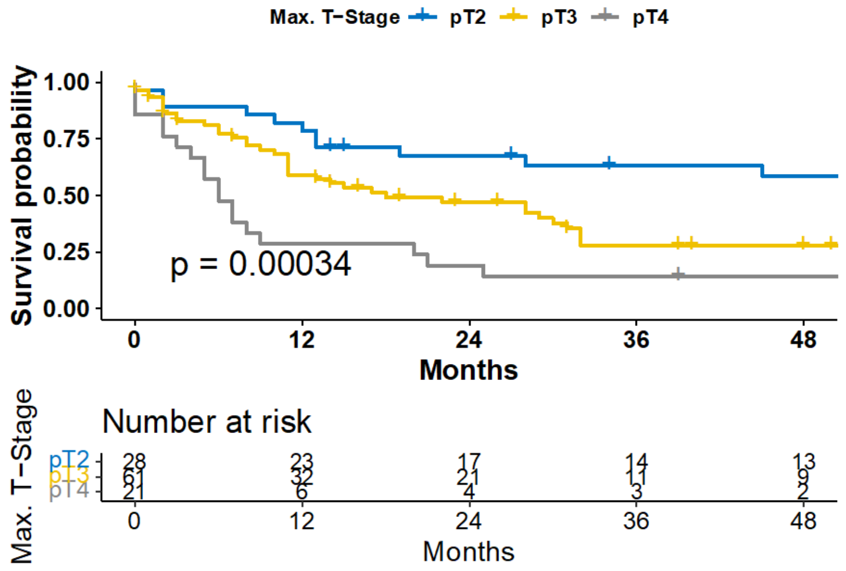

Figure 1: Kaplan-Meier curve for overall survival for all patients stratified by pathological tumor stage.

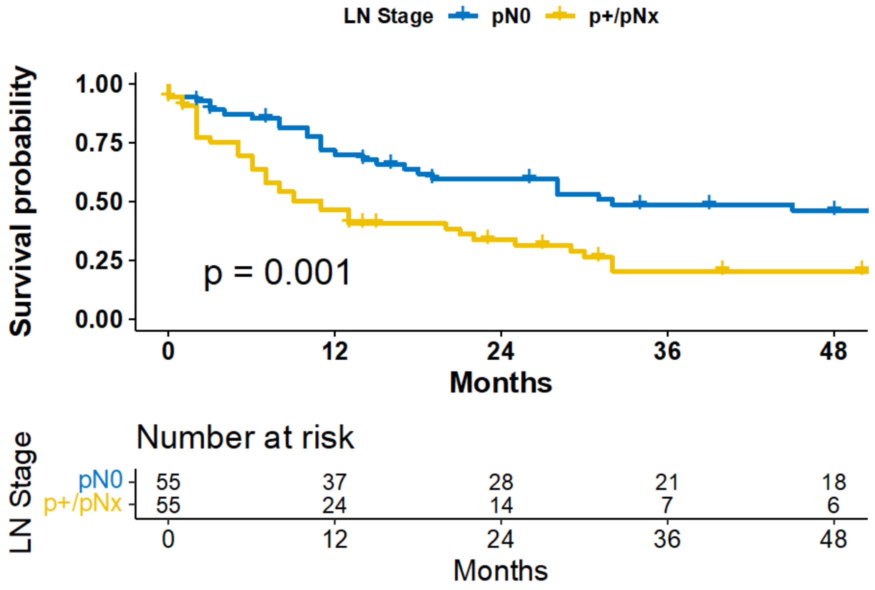

Figure 2: Kaplan-Meier curve for overall survival for all patients stratified by lymph node status

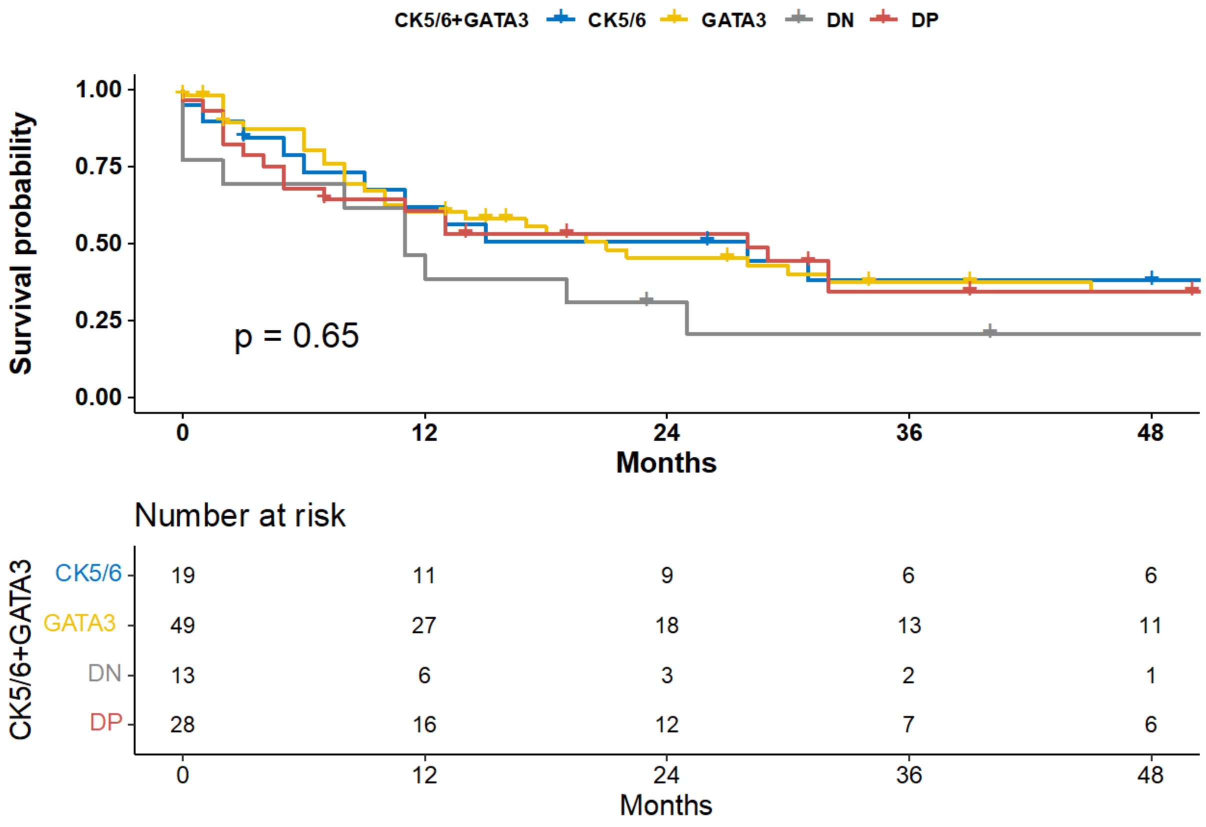

Figure 3: Kaplan-Meier curve for overall survival for patients stratified by IHC markers. DN = double negative; DP = double positive

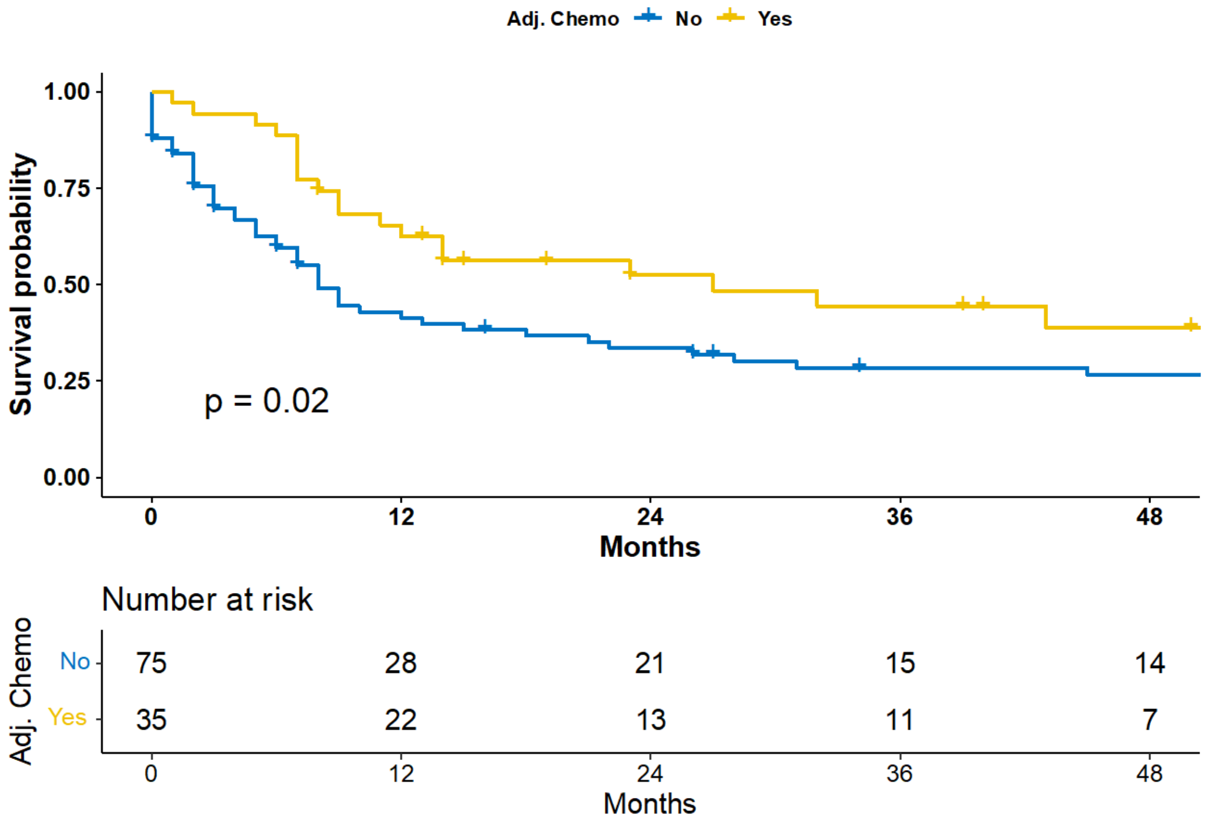

Figure 4: Kaplan-Meier curve for disease-free survival for patients with and without adjuvant chemotherapy.

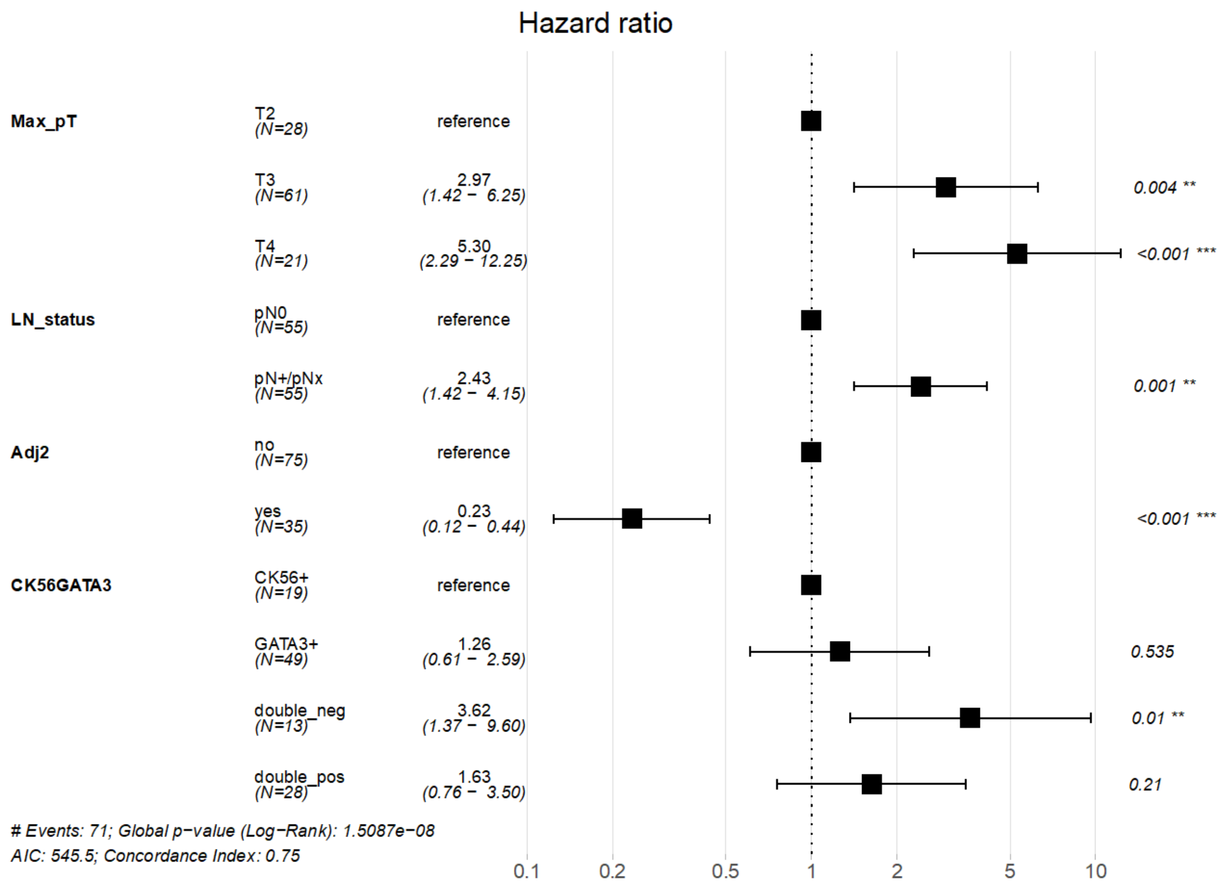

Figure 5: Multivariate cox-regression model for disease-free survival adjusting for tumor and LN stage, adjuvant chemotherapy, and the IHC-markers CK5/6 and GATA3. LN = lymph node; Adj2 = at least two cycles of adjuvant chemotherapy
